# Genotype–Phenotype correlations of SCARB2 associated clinical presentation: a case report and in-depth literature review

**DOI:** 10.1186/s12883-022-02628-y

**Published:** 2022-03-28

**Authors:** Burcu Atasu, Ayse Nur Ozdag Acarlı, Basar Bilgic, Betül Baykan, Erol Demir, Yasemin Ozluk, Aydin Turkmen, Ann-Kathrin Hauser, Gamze Guven, Hasmet Hanagasi, Hakan Gurvit, Murat Emre, Thomas Gasser, Ebba Lohmann

**Affiliations:** 1 Hertie Institute for Clinical Brain Research, University of Tübingen, Tübingen, Germany; 2 German Center for Neurodegenerative Diseases (DZNE)-Tübingen, Tübingen, Germany; 3 Department of Neurology, Istanbul Faculty of Medicine, Istanbul University, Istanbul, Turkey; 4 Division of Nephrology, Department of Internal Medicine, Istanbul Faculty of Medicine, Istanbul University, Istanbul, Turkey; 5 Department of Pathology, Istanbul Faculty of Medicine, Istanbul University, Istanbul, Turkey; 6 Division of Nephrology, Department of Internal Medicine, Koc School of Medicine, Koc University, Istanbul, Turkey; 7 Institute for Experimental Medicine, Genetics Department, Istanbul University, Istanbul, Turkey

**Keywords:** SCARB2, Action Myoclonus-Renal Failure Syndrome, Progressive Myoclonic Epilepsy, Gaucher disease, Parkinson’s disease, Ataxia, Pathogenic variants

## Abstract

**Background:**

Biallelic pathogenic variants in the *SCARB2* gene have been associated with action myoclonus-renal failure (AMRF) syndrome. Even though *SCARB2* associated phenotype has been reported to include typical neurological characteristics, depending on the localization and the feature of the pathogenic variants, clinical course and the presentations have been shown to differ.

**Case presentation:**

Whole exome sequencing (WES) analysis revealed a homozygous truncating variant (p.N45MfsX88) in *SCARB2* gene in the index case, and subsequent sanger sequencing analysis validated the variant in all affected family members from a Turkish family with the clinical characteristics associated with AMRF and related disorders. Intrafamilial clinical heterogeneity with common features including dysarthria, tremor and proteinuria, and distinct features such as peripheral neuropathy (PNP), myoclonus and seizures between the affected cases, was observed in the family.

In-depth literature review enabled the detailed investigation of the reported variants associated with AMRF and suggested that while the type of the variant did not have a major impact on the course of the clinical characteristics, only the C terminal localization of the pathogenic variant significantly affected the clinical presentation, particularly the age at onset (AO) of the disease.

**Conclusions:**

In this study we showed that biallelic *SCARB2* pathogenic variants might cause a spectrum of common and distinct features associated with AMRF. Of those features while the common features include myoclonus (100%), ataxia (96%), tonic clonic seizures (82%), dysarthria (68%), tremor (65%), and renal impairment (62%), the uncommon features involve PNP (17%), hearing loss (6.8%), and cognitive impairment (13.7%). AO has been found to be significantly higher in the carriers of the p.G462DfsX34 pathogenic variant.

*SCARB2* pathogenic variants have not been only implicated in AMRF but also in the pathogenesis of Parkinson’s disease (PD) and Gaucher disease (GD), suggesting the importance of genetic and functional studies in the clinical and the diagnostic settings.

Given the proven role of *SCARB2* gene in the pathogenesis of AMRF, PD and GD with a wide spectrum of clinical symptoms, investigation of the possible modifiers, such as progranulin and HSP7, has a great importance.

**Supplementary Information:**

The online version contains supplementary material available at 10.1186/s12883-022-02628-y.

## Background

Action myoclonus-renal failure syndrome (AMRF; MIM 254,900) is a rare autosomal recessive inherited disorder with progressive neurological and renal symptoms. The disease usually presents in the first two decade of the life, and some patients do not develop renal failure in the course of the disease. Neurologic manifestations can appear before, simultaneously, or after the renal manifestations and include myoclonus, epileptic seizures, ataxia, tremor, peripheral neuropathy, hearing loss and behavioral problem, whereas renal symptoms consist of proteinuria and kidney failure. AMRF was first described in four French Canadian patients whose symptoms were myoclonus, cerebellar symptoms, and proteinuria [1, 2]. Recently, loss-of-function pathogenic variants in *scavenger receptor class B member 2* gene (*SCARB2*), which encodes lysosomal integral membrane protein type 2 (LIMP-2) protein [3, 4] have been identified in AMRF patients [5]. LIMP-2 is an abundant, highly glycosylated lysosomal membrane protein that plays a pivotal role in the delivery of β-glucocerebrosidase (GC) to lysosomes [4]. GC is required for lysosomal hydrolysis of glucosylceramide and is targeted to lysosomes in a mannose-6 phosphate-independent manner by LIMP-2 [6]. LIMP-2 and GC interact in the endoplasmic reticulum (ER), and the two proteins traverse Golgi and endocytic compartments together en route to lysosomes, at which the acidic pH facilitates dissociation of GC from LIMP-2 [4, 6]. *SCARB2* pathogenic variants lead to accumulation of the LIMP-2 protein in the ER, thereby leading to missorting and lysosomal depletion of GC [6]. Decreased lysosomal activity of GC is also seen in GD, the most common lysosomal storage disease caused by the homozygous pathogenic variants in the gene encoding GC, as well as in PD, of which *SCARB2* is a risk conferring gene. Deficient activity of GC was observed in cultured skin fibroblasts of AMRF patients [7].

*SCARB2* associated phenotype has been shown to include a wide spectrum of neurological characteristics with varying AO depending on the localization of the pathogenic variants. (Table 2). By taking the advantage of WES strategy, in this study we investigated the underlying genetic cause of a consanguineous Turkish family with five siblings presenting with neurological and renal symptoms, compared our findings to the literature and developed a genotype–phenotype correlation based on the reported cases with SCARB2 pathogenic variants.

## Case presentation

**Individual II.8** is a 31-year-old male with normal motor and mental development and had no medical problems until the age of 22 years when he developed speech problems and behavioral changes such as irritability and depressed mood. At the age of 24 years tremor in his hands and mild gait instability added to the clinical picture. One year later myoclonic jerks triggered by noise, mental tasks and stress and generalized tonic–clonic seizures appeared. At the age of 26 years proteinuria was detected in his routine exams. He was admitted to the Outpatient Clinic of Neurology Department of Istanbul University when he was 28 years old. In his neurological examination, significant myoclonic movements in his limbs, trunk, and the lower facial muscles mostly appearing with the movements of the limbs were observed, and jerks were sensitive to touch. There were also jerky movements with a relatively small amplitude in the rest position. He had hesitations of speech and dysarthria due to bulbar and palatal myoclonus. He presented prominent gait instability due to ataxia and high amplitude myoclonic movements (see supplementary file, Video_1.mov and Video_2.mov). Cognitive testing showed impairment in executive functions and attention. The patient was depressed (Beck’s Depression Inventory 21/30). Serum creatinine was in a normal range and 24-h urine protein level was 3540 mg. Magnetic resonance imaging (MRI) of the brain and peripheral nerve conduction velocity (NCV) were normal. Long latency reflexes and giant somatosensory evoked potentials (SEP) were recorded. The electroencephalography (EEG) findings showed 3–4 Hz generalized epileptiform abnormalities, a broad range of photosensitivity (during 5–24 Hz intermittent photic stimulations), as well as diffuse background slowing of theta range. Ictal spike-wave discharges were recorded corresponding to the myoclonic jerks. His audiometry was normal. Abdominal ultrasound showed increased cortical echogenicity of the kidneys bilaterally (suggesting renal parenchymal disease) and mild splenomegaly.

Kidney biopsy showed nine glomeruli, one of which was globally sclerotic. Segmental sclerotic lesion was observed in one glomerulus, and immunofluorescence microscopy demonstrated segmental deposition of IgM in capillary loops. Biopsy findings were compatible with a diagnosis of focal segmental glomerulosclerosis (FSGS). Collapsing features of segmental sclerosis were partially present. Transmission electron microscopic evaluation was demonstrated food process effacement with osmophilic irregular conglomerate electron-dense inclusions within endothelial and tubular cell cytoplasm. Some inclusions were filamentous, lamellar, and rectilinear in structure.

He was put on zonisamide (200 mg/day), piracetam (9600 mg/day), clonazepam (2 mg/day) and losartan (100 mg/day). Antiepileptic drugs had a limited beneficial effect on generalized seizures. However myoclonic movements did not respond to treatment. The patient stopped all his treatments at the age of 29 except for losartan. His symptoms worsened over time, and he accepted to use Miglustat (300 mg/day), but after a month he discontinued the treatment due to severe exacerbation of action myoclonus and ataxia.

Initial symptoms of the **individual II.9** were irritability, speech impairment, tremor, and myoclonic movements started at the age of 21 years. Two years later cerebellar ataxia and generalized seizures added, and he admitted for the first time to the neurology department when he was 27 years old. Neurological examination revealed dysarthria with speech induced bulbar myoclonus and there were severe myoclonic movements in his distal parts of the limbs as well as in his trunk. Myoclonic jerks were significant especially when he was moving his upper extremities and when he was speaking. However, some jerky movements were also present in the resting position. Gait was severely impaired by both vigorous myoclonic movements and cerebellar involvement. Although the patient had no cognitive complaints, the neuropsychological assessment showed impairment in executive functions and attention, similar to his elder brother (individual II.8). Results of routine biochemistry were in the normal limits. The 24-h urine protein level was 336 mg/day. MRI of the brain and whole medulla spinalis revealed normal. Peripheral nerve conduction velocity was normal, but long latency reflexes were recorded in the SEP. The EEG showed generalized epileptiform abnormalities as well as diffuse slowing of the background activity. His audiometry was normal. He was treated with valproic acid (maximum dose 2000 mg/day), levetiracetam (maximum dose 2500 mg/day), piracetam (maximum dose 4800 mg/day) and clonazepam (maximum dose 3 mg/day) but no significant effect was noticed. Myoclonic movements and ataxia worsened overtime, and he became bedridden. He developed dysphagia which led to aspiration pneumonia, and he died because of septicemia when he was 28 years old.

**Individual II.10** was complaining about mild and rare twitches in the perioral muscles, and mild tremor in his hand started a year ago. In his neurological examination he had mild dysarthria, and there were rare speech-induced myoclonic movements in his bulbar muscles as well as mild tremor in his hands. He had no other myoclonic movements and no ataxia was observed. Serum and urine laboratory results were in normal limits except for proteinuria (515 mg/day). Brain MRI and electrophysiological examinations including peripheral NCV and SEP were normal. Concentric needle electromyography (EMG) of the proximal and distal muscles showed large-amplitude polyphasic motor unit potentials and widespread decreased recruitment of motor units without spontaneous activity. No epileptiform activity or slowing was seen in his EEG.

Mild myoclonic movements appeared in **individual II.12** when he was 8 years old. At the age of 16, generalized seizures along with tremor, and speech problems were added to the clinical picture and two years later he developed ataxia. He admitted to our department when he was 21 years old, and in his neurological examination, there were dysarthria and ataxia, and myoclonic movements worsening with motor activity in his perioral muscles and extremities. He had also increased levels of urinary protein (500 mg/day). MRI of the brain was normal. Although the peripheral NCV was in the normal limits, concentric needle EMG of the proximal and distal muscles showed large-amplitude polyphasic motor unit potentials and widespread decreased recruitment of motor units without spontaneous activity. In his SEP examination, stimulation of the median nerve elicited normal responses however prolonged latency of the cortical P37 was recorded with the tibial nerve stimulation. The EEG showed no abnormality, and his audiometric evaluation was normal. Abdominal ultrasound examination showed findings of low-grade fatty liver with increased liver echogenicity. He was treated with valproic acid (maximum dose 1000 mg/day), and levetiracetam (maximum dose 1000 mg/day). He was put on Miglustat (100 mg/day), but in the first week of the treatment, he refused to use the drug due to worsening of myoclonic movements and ataxia.

**Individual II.13** was admitted to the hospital with complaints of typhoid fever. Nephrotic range proteinuria (9631 mg/day) was detected in his laboratory results. He had no myoclonic movements, ataxia or epileptic seizures. He underwent a renal biopsy, and it showed FSGS. Immunofluorescence microscopy showed deposition of one positive C1q, C3, and IgM in capillary loops. Even he had no neurological complaint, at the time of the assessment of all family members, it was noticed that he had mild difficulty in speech and mild tremor in his hands. Sensory loss and impaired vibration sensation were noticed in the distal part of the extremities with normal deep tendon reflexes. The EMG revealed a mild axonal type of sensorimotor neuropathy. Serum vitamin B12 level was found to be decreased, and he was put on replacement treatment. The EEG and cranial MRI revealed normal.

In this study, consistent with the previously reported renal biopsy findings renal biopsy showed tubular abnormalities in the distal and collecting tubules with isometric vacuolization. In addition, there were FSGS (with collapsing glomerulopathy) and the deposition of granular material in cortical tubules, and glomeruli without inflammatory infiltration. These findings were compatible with FSGS with non-specific immunoglobulin and complement trap. There was osmophilic irregular conglomerate electron-dense inclusions in the glomerulus in the individual II.8, which could be further investigated to provide with more insight into the renal involvement of the disease.

**Fig. 1:**
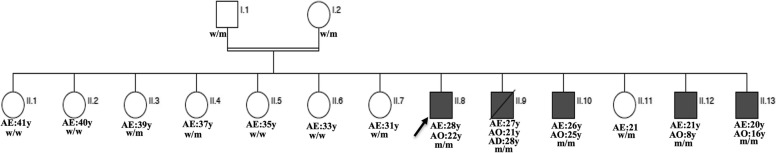
Pedigree of the family 1. AO: age at onset, AE: age at examination, AD: age at death, y: years, w: wild-type allele, m: mutant-allele (p.N45MfsX88)

To uncover the genetic cause of the clinical presentation observed in the family 1, WES analysis was performed in the index case (II.8) by following the same workflow described elsewhere [8]. Consistent with the pedigree information inbreeding coefficient (F) was found to be “0.1162” (for a first cousin marriage F ≥ 0.0625), confirming the consanguinity in the family. WES analysis revealed a homozygous truncating variant (p.N45MfsX88) in *SCARB2* gene in the index case (II.8). Subsequent Sanger sequencing analysis identified the same homozygous variant in the other affected individuals (II.9, II.10, II.12, II.13), whereas all unaffected family members were either heterozygot (I.1, I.2 II.3, II.4, II.7, II.11) or non-carrier (II.1, II.2, II.5, II.6) (Fig. 1).

### Summary and clinical comparison of the cases with SCARB2 p.N45MfsX88 pathogenic variant

In this study, we describe a multiple affected (*n* = 5) consanguineous Turkish family with a homozygous truncating pathogenic variant (c.134delA**;** p.N45MfsX88), which has been recently reported in an independent Turkish family [9] (family 2) in the *SCARB2* gene presenting with similar clinical presentation with slightly different AO of the disease and without dysarthria and cognitive impairment (Table 1).

**Table 1:** Clinical characteristics of the cases with the SCARB2 p.N45MfsX88 Pathogenic Variant

**ID**	**Gender**	**Age at onset of symptoms (years)**					**Deceased**		**Imaging**	**EEG**	**EMG**	**Mental status**
		**Dysarthria**	**Tremor**	**Myoclonus**	**Seizures**	**Renal Involvement**	**Age (y)**	**Cause**				
Family 1												
II.8	M	22	24	25	25	26	-		Normal brain MRI	BGS, PS, GSW	Normal NCV Long latency reflexes and giant SEP	Impaired executive functions/ attention
II.9	M	21	21	21	23	27	28	Aspiration pneumonia	Normal brain and spinal MRI	BGS, ECS, GSW	Normal NCV Long latency reflexes	Irritability, impaired executive functions/ attention
II.10	M	25	25	-	-	25	-		Normal brain MRI	Normal	Normal NCV/ SEP, Polyphasic MUPs and widespread decreased recruitment of motor units without spontaneous activity	NR
II.12	M	19	17	8	16	19	-		Normal brain MRI	Normal	Normal NCV/ SEP, Polyphasic MUPs and widespread decreased recruitment of motor units without spontaneous activity	NR
II.13	M	20	20	-	-	16	-		Normal brain MRI	Normal	Sensorimotor axonal neuropathy	NR
Family 2												
Case 1	M	NR	17–18	18–19	18–19	+	-		Normal brain MRI	GSW	Sensorimotor axonal neuropathy	Normal
Case 2	F	NR	23	24	24	+	-		Normal brain MRI	GSW	-	Normal

This table indicates the clinical characteristics of the family 1 and the family 2 [9] harboring the same pathogenic variant (p.N45MfsX88) reported from Turkey. Family 1 is described in this study and family 2 was recently published by Uçan Tokuç et al. [9]. *BGS* background slowing, *PS* photosensitivity, *GSW* generalized spike-wave, *ECS* eye closure sensitivity, *NCV *nerve conduction velocity, *SEP* somatosensory evoked potential, *MUP* motor unit potential, *y* years, *M* male, *F* female, *NR* not reported, *MRI* magnetic resonance imaging

In the family 1 the cardinal clinical features shared in all the affected individuals are dysarthria, tremor and proteinuria, other features such as PNP, myoclonus and seizures are only observed in some of the affected members (Table 1). In general, all the clinical features except for dysarthria and cognitive decline are shown to be comparable with the second family also originating from Turkey and harboring the same pathogenic variant (Table 1). This report also shows that the same *SCARB2* pathogenic variant can cause intra-clinical heterogeneity including different AO of the symptoms having a range from 8 to 25 years.

Neuropsychological testing revealed executive dysfunction and attention deficit in two patients (II.8 and II.9) of family 1, however, no cognitive dysfunction was reported in the family 2.

### Literature review of reported cases with SCARB2 pathogenic variants

The Medline and the human mutation database [10] were searched using terms as *SCARB2*, *CD36L2* and *LIMP2*. All the reported pathogenic variants in *SCARB2* gene were reviewed in terms of clinical and genetic characteristics. We classified the variants based upon if those have been associated with AMRF (Table 2) or not directly AMRF (Table 3).

**Table 2:** Clinical and Genetic Characteristics of the Reported AMRF-Associated Families

Genetic findings				Clinical characteristics												
**Type of the variant**	**Pathogenic Variant**	**Zygosity**	**Localization on the protein**	**Age at onset**^**a**^	**Age at death**^**b**^	**Myoclonus**^**a**^	**Tremor**^**a**^	**Ataxia**^**a**^	**Dysarthria**^**a**^	**Seizure**	**Renal involvement/ FSGS**^**a**^	**PNP**	**Additional clinical manifastations**	**Reported families/ affected individuals**	**Etnicity**	**Ref**
Nonsense	c.533G > A; p.W178X	HM	GC binding domain	16 ± 1.4 (15, 17)	25.5 ± 2.1 (23, 26)	16 ± 1.4 (15, 17)	NR	17.5 ± 0.7 (15, 18)	+	NR	19.5 ± 2.1 (18, 21)/-	-	Slowed horizontal saccadic eye movements	1/2	Portuguese	[7]
Splicing	c.1239 + 1G > T	HM	Lumenal domain	22	NR	32	25	32	32	NR	28/ +	-	NR	1/1	Turkish-Cypriot	[5]
Frameshift	c.435_436insAG; p.W146SfsX16^c^	HM	Lumenal domain	9	32	29	21	+	+	+	9/ +	NR	NR	1/1	Scottish	[5]
Frameshift/ Splicing	c.296 delA; p.N99IfsX34 /c.704 + 5G > A	CH	Lumenal domain/ Lumenal domain	19	NR	24	19	26	26	+	20/NR	NR	NR	1/1	British	[5]
Nonsense	c.862C > T; p.Q288X^c^	HM	GC binding domain	20 ± 4.4 (17, 18, 25)	29.7 ± 5 (29, 25, 35)	23 ± 2 (21, 23, 25)	20 ± 4.4 (17, 18, 25)	+	+	+	22.5 ± 0.7/NR (17, 18, 30)	NR	NR	1/3	French Canadian	[5]
Missense	c.1087C > A; p.H363N	H^f^	Lumenal domain	26	NR	26	NR	27	NR	+	-	-	NR	1/1	NR	[11]
Splicing	c.1116‑2A > C	HM	Lumenal domain	14	29	14	NR	17	NR	+	15/NR	NR	NR	1/1	Italian	[12]
Splicing	c.704 + 1G > C	HM	Lumenal domain	15	27	15	NR	16	NR	+	12/NR	NR	NR	1/1	Italian	[12]
Frameshift	c.1258delG; p.E420RfsX5	HM	Lumenal domain	23	33	23	NR	24	NR	+	10/NR	NR	NR	1/1	Italian	[12]
Frameshift	c.666delCCTTA; p.Y222X	HM	GC binding domain	25	40	25	NR	31	NR	+	15/NR	NR	NR	1/1	Italian	[12]
Splicing/ Missense	c.424‑2A > C/ c.1087C > A; p.H363N	CH	Lumenal domain/ Lumenal domain	5.5	NR	26	26	26	NR	+	5.5/NR	NR	NR	1/1	Italian	[12]
Nonsense/ Frameshift	c.862C > T; p.Q288X^c^/ c.1187 + 3insT	CH	GC binding domain/ Lumenal domain	16	NR	16	NR	NR	+	+	-	+	NR	1/1	French Canadian	[13]
Frameshift	c.111delC; p.I37MfsX7	HM	Lumenal domain	18 ± 3.6 (14, 20, 20)	34.3 ± 3.5 (31, 34, 38)	20 ± 6 (14, 20, 26)	NR	18 ± 3.6 (14, 20, 20)	NR	+	+ /NR	+	Hearing impairment, dilated cardiomyopathy	1/3	German	[14]
Splicing	c.704 + 1G > A	HM	Lumenal domain	21	NR	23	23	21	25	+	25/NR	NR	Hearing loss	1/1	Australian	[15]
Frameshift	c.1015insT; F339FfsX9	HM	Lumenal domain	22	NR	22	22	30	30	NR	-	NR	Slowed saccades, myoclonic status	1/1	NR	[16]
Frameshift	c.1385_1390delinsATGCATGCACC; p.G462DfsX34	HM	TM domain	46.6 ± 4 .7 (43, 52, 45)	+ (NR, NR, 59)	52 ± 13.8 (43, 68, 45)	+ (NR, 57, 48)^e^	48.3 ± 4 (44, 52, 49)	50.6 ± 6.5 (44, 57, 51)	+ (58, 63, NR)^e^	-/NR	NR	Dementia [17]	2/3	Japanese	[17, 18]
Nonsense	c.361C > T; p.R121X	HM	Lumenal domain	20	28	20	+	+	+	+	-	-	Cognitive impairment	1/1	Japanese	[18]
Nonsense	c.1270C > T; p.R424X	HM	Lumenal domain	20.5 ± 4.7 (17, 17, 21, 27)	+ (17, 17, NR, 27–34)	+	+	+	+	+	-/NR	NR	Mild generalized skeletal muscle atrophy (34)	2/4	Arab [19], NR [20]	[19, 20]
Splicing	c.995‑1G > A	HM	Lumenal domain	20.5 ± 0.7 (21, 20)	NR	20.5 ± 0.7 (21, 20)	NR	20.5 ± 0.7 (21, 20)	+	+	-	NR	Pes cavus, Mild generalized skeletal muscle atrophy	1/2	Chinese	[21]
Splicing	c.1187 + 5G > T	HM	Lumenal domain	19	NR	19	19	19	+	+	-	+	NR	1/1	Chinese	[22]
Frameshift	p.L14PfsX35	HM	TM domain	19	NR	19	+	27	27	+	+ /NR	NR	NR	1/1	Arab	[23]
Frameshift	c.134delA; p.N45MfsX88	HM	Lumenal domain	20 ± 4.2 (17, 23)	NR	21.5 ± 3.5 (19, 24)	20 ± 4.2 (17, 23)	+	NR	+	+ /NR	+	NR	1/2	Turkish	[9]
Frameshift/ Splicing	c.434_435dup?/ c.704 + 5G > A	CH	Lumenal domain/ Lumenal domain	19	NR	19	19	+	NR	-	+ /NR	NR	NR	1/1	British or Irish	[24]
Frameshift	p.L31RfsX6	HM	Lumenal domain	27	NR	27	+	30	30	-	-	NR	NR	1/1	Gambian	[25]
Splicing	c.423 + 1G > A	HM	Lumenal domain	20.5 ± 2.1^d^ (22, 19)	NR	32 ± 2.8 ^d^ (34, 30)	20.5 ± 2.1^d^ (22, 19)	33 ± 1.4 ^d^ (34, 32)	40.5 ± 6.3 ^d^ (36,45)	+	40.5 ± 6.3 ^d^ (36,45) /NR	NR	NR	1/6	Iranian	[26]
Frameshift /Nonsense	c.435_436insAG; p.W146SfsX16^c^/ c.862C > T; p.Q288X^c^	CH	Lumenal domain / GC binding domain	20	NR	23	NR	23	22–23	20	±	NR	Unilateral ureteropelvic junction	1/1	French Canadian/ Irish English	[27]
Frameshift	c.134delA**;** p.N45MfsX88	HM	Lumenal domain	18.4 ± 6.7	28 (II.9)	18 ± 8.9	21.4 ± 3.2	19.9 ± 2.6	21.4 ± 2.3	+	28 (II.9)/ +	21 (II.13)	Irritability, impaired executive functions/ attention	1/5	Turkish	Present study

This table indicates the genetic and clinical features of the reported families associated with AMRF. ^a^Average age at onset was indicated, in case of more than one carriers of the same mutation have different ages at onset. In case of only one affected from a family, age at onset of symptoms corresponding to that individual was indicated. When available, age at onsets of all the individuals were indicated in parenthesis. ^b^Average age at death was indicated, in case of more than one carriers of the same mutation have different ages at death. When available, age at deaths of all the individuals were indicated in parenthesis. ^c^Founder mutation. ^d^The average age at onsets were indicated based on the clinical information of two reported individuals (IV.9, IV.12). ^e^When the average age at onsets could not be calculated, only the onsets of the corresponding symptoms for each individual were indicated. ^f^The possibility of second SCARB2 PV. NR: not reported in the corresponding study, -: not observed in the corresponding study, *HM* homozygote, *CH* compound heterozygot, *H* heterozygot, + : observed in the patients but not detailed information provided in the corresponding studies, ?: the correct nomenclature could not be found in the original study

**Table 3:** Clinical and Genetic Characteristics of the non-AMRF Associated Families

Genetic findings				Clinical characteristics	Implication of the Variant	Reported families/ affected individuals	Reference
**Type of the variant**	**Pathogenic Variant**	**Zygosity**	**Localization on the protein**				
Missense; de novo	c.518 T > C;p.V173A	H	GC binding domain	ASD	Implication in chromatin modification, FMRP-associated mechanisms and embryonic development	1/1	[28]
Missense	c.842 T > C;p.F281S	H	GC binding domain	SUDEP	Implication in the underlying mechanisms of SUDEP	1/1	[29]
Missense	c.914C > T;p.Y305M	H	Lumenal domain	PS	Possible modifier in the PS	1/2	[30]
Nonsense	c.1365; p.W455X	CH	Lumenal domain	Epilepsy and neurodevelopmental disorders	Implication in the underlying mechanisms of epilepsy and neurodevelopmental disorders	1/1	[31]
Frameshift	c.434_435insAG; p.W146SfsX16^a^	CH	Lumenal domain				
Missense	c.1412A > G;p.E471G	H	TM domain	Gaucher disease, myoclonic epilepsy, dementia	Possible modifier in the binding dependent mechanisms of LIMP-2 and GC	1/2	[32]
Frameshift	c.350_351delAT; p.Y117CfsX3	HM	Lumenal domain	Cerabellar ataxia, seizures, myoclonus and dementia reported in general. Detailed patient specific information was NR	Implication in the cerabellar ataxia, seizures, myoclonus and dementia	1/1	[33]
Frameshift	c.1337delG;p.G446VfsX48	CH	Lumenal domain	Seizures	NR	1/1	[34]
Frameshift	c.434_435insAG;p.W146SfsX16^a^	CH	Lumenal domain				

Molecular and clinical details of the reported pathogenic variants associated with atypical clinical features. ^a^the variant was associated with AMRF in a homozygous state. *NR* Not reported in the corresponding study, *HM* homozygote, *CH* compound heterozygot, *H* heterozygot, *ASD* autism spectrum disorder, *SUDEP* sudden unexpected death in epilepsy, *PS* Pendred syndrome

### SCARB2 pathogenic variants associated with AMRF

To date, 26 distinct pathogenic variants including nonsense (*n* = 4), splice site (*n* = 9), frameshift (*n* = 12), missense (*n* = 1) variants, have been reported in 48 cases from 29 independent families with AMRF from various ethnic groups. The mean AO and age at death were found to be 19.4 ± 6.9 and 30.6 ± 2.3, respectively. The clinical and the genetic characteristics of the reported families were summarized in the Table 2. The families have been reported from various ethnic groups (*n* = 1 Portugese, *n* = 1 Turkish-Cypriot, *n* = 1 Scottish, *n* = 2 British, *n* = 3 French-Canadian, *n* = 5 Italian, *n* = 1 German, *n* = 1 Australian, *n* = 3 Japanese, *n* = 2 Arab, *n* = 2 Chinese, *n* = 2 Turkish, *n* = 1 Gambian, *n* = 1 Iranian). No population specific pathogenic variance distribution was observed.

Two of the previously reported pathogenic variants (p.Q288X; p.W146SfsX16) have been shown to be founder variants in French-Canadian and Scottish populations [5, 13]. So far, only one pathogenic variant (p.N45MfsX88) in *SCARB2* has been identified in the Turkish population besides the variant (c.1239 + 1G > T) found in the Turkish-Cypriot population [5]. Therefore, it is highly possible that this particular variant (c.134delA**;** p.N45MfsX88) be a founder variant and this possibility could be further investigated by haplotype analysis.

The common clinical features observed in the AMRF patients harboring SCARB2 pathogenic variants include myoclonus (*n* = 29), ataxia (*n* = 28), tonic clonic seizures (*n* = 24), dysarthria (*n* = 20), tremor (*n* = 19) and renal impairment (*n* = 18), whereas the uncommon features of those involve PNP (*n* = 5), hearing loss (*n* = 2), and cognitive impairment (*n* = 4).

The statistical analysis showed that only the C terminal localization of the pathogenic variant is significantly associated with the AO (Mann–Whitney U test, *P* < 0.001), however, neither the type of the pathogenic variant nor the domain localization of the pathogenic variant is associated with AO or age at death (Mann–Whitney U test, *P* > 0.05).

Cognitive decline has been reported only in four AMRF cases from three unrelated families [17, 18].

### SCARB2 pathogenic variants associated with non-AMRF

*SCARB2* variants have also been implicated in differential clinical conditions overlapping or not with the symptoms of AMRF (Table 3). In addition to the AMRF associated variants, seven distinct *SCARB2* variants, including four missense, one nonsense, and two frameshift variants, have been identified. All the missense variants have been found in a heterozygous state while, nonsense and frameshift variants have been found either in homozygous or compound heterozygous state. Interestingly, two heterozygous variants (c.914C > T;p.Y305M, c.1412A > G;p.E471G) have been reported to be possible modifiers in the Pendred syndrome (PS) [30], which is characterized by the combination of sensorineural deafness/hearing impairment, goiter, and an abnormal organification of iodide with or without hypothyroidism, and in GD pathogenesis [32]. In addition, two other variants (c.518 T > C;p.V173A, c.842 T > C;p.F281S) have been suggested to be risk conferring variants in autism spectrum disorder [28] and sudden unexpected death in epilepsy [29]. Strikingly, of those variants, p.E471G was proposed as a possible indicator of the convergent disease mechanisms of the AMRF and GD. The remaining biallelic nonsense and frameshift variants were associated with the spectrum of AMRF symptoms, but their phenotype only included seizures, myoclonus, or cerebellar ataxia, suggesting the importance of the WES based approaches in the clinical-diagnostic settings.

## Discussion and conclusions

**Fig. 2:**
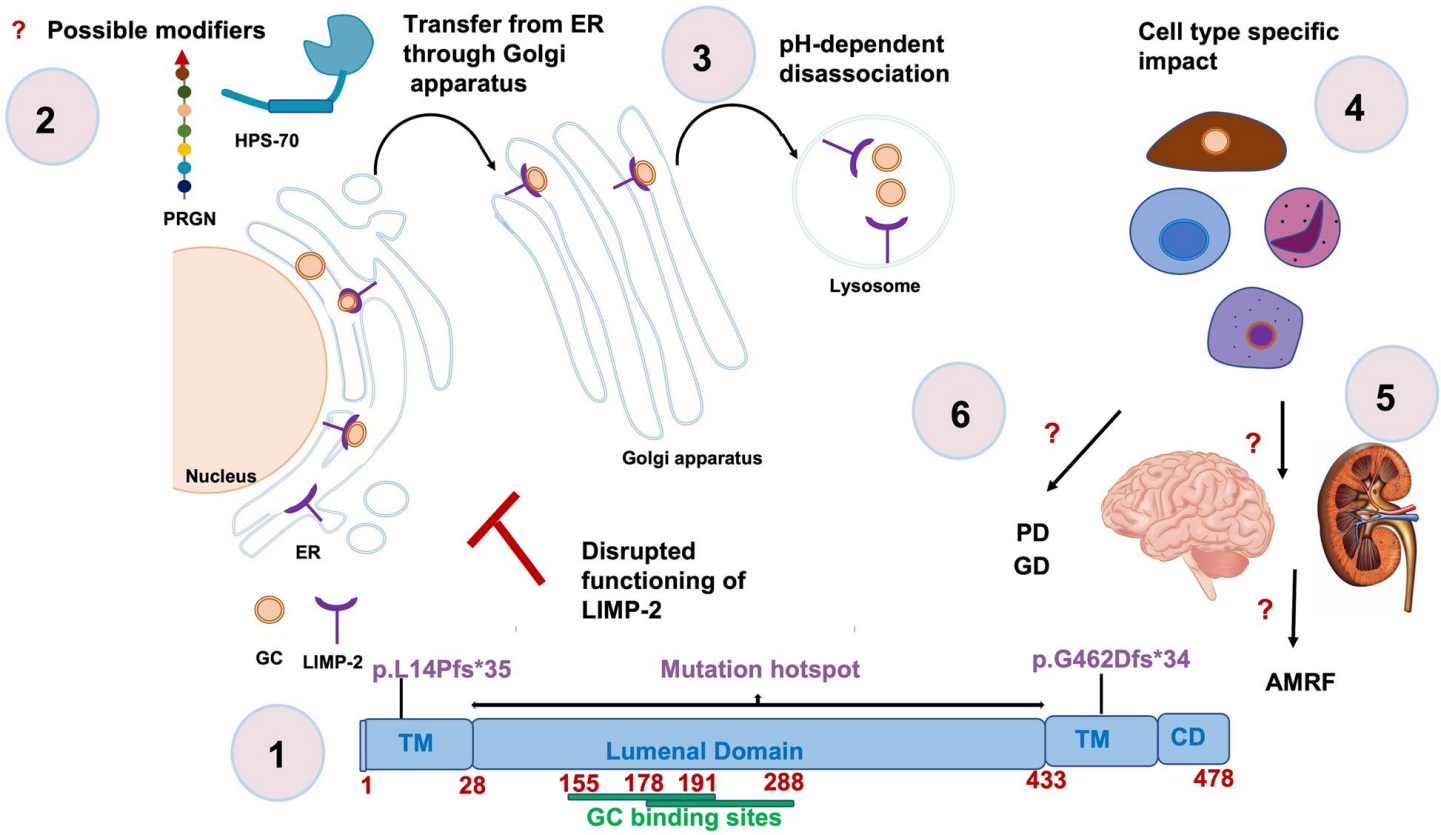
Characteristics of the LIMP-2 associated mechanisms. 1-) Illustration of the LIMP-2 active domains and the particular mutation locations. 2-) LIMP-2 and GC complex formation, transfer of LIMP-2 and GC complex form ER through Golgi apparatus, and possible modifiers implicated in those molecular mechanisms. Cellular models with *SCARB2* pathogenic variants showed ER retention of LIMP-2 independent from the disturbed affinity of the GC binding site. 3-) Disassociation of the LIMP-2 and GC complex in a pH-dependent manner in lysosomes. 4-) Unexplained cell type specific impact of the perturbed mechanisms. 5-) Unexpained mechanisms causing disfunction of brain and kidney in AMRF. 6-) Convergent mechanisms implicated in PD, GD and AMRF. GC: β-glucocerebrosidase, TM: transmembrane domain, CT: cytoplasmic domain, ER: endoplasmic reticulum, AMRF: action myoclonus-renal failure, PD: Parkinson's disease, GD: Gaucher disease

Biallelic pathogenic variants in *SCARB2* gene have been shown to cause AMRF with a wide spectrum of neurological and renal symptoms. *SCARB2* gene encodes a type III transmembrane glycoprotein called LIMP-2. LIMP-2 consists of four domains, of which three are cytoplasmic (CT) and transmembrane (TM) domains, and one lumenal domain (residues 28–433) encompassing the GC binding site (residues 155–191, 178–288) [6] functioning in a pH dependent manner (Fig. 2–1). Genetic as well as the functional studies imply that disturbed LIMP-2 activity causes perturbed trafficking of GC and thereby leads to the retention of LIMP-2 in the ER and/or secretion to the extracellular space suggesting impaired targeting of the GC to the lysosomes (Fig. 2–3), which is also a pathomechanism implicated in GD and PD [35]. Investigation of the function of the LIMP-2 is therefore highly important in terms of shedding light on the convergent pathogenic mechanisms of the neurological diseases, such as AMRF, GD and PD (Fig. 2–6).

To date, different type of variants from various ethnic groups have been reported to be associated with AMRF. Interestingly, except for only one missense variant (p.H363N), of all the reported variants are loss of function variants (*n* = 25), which are supposed to lead to a truncation or total absence of the protein through nonsense mRNA-mediated decay in the cellular systems. However, even though the reported missense variant is not expected to cause such a harmful effect, the clinical picture of the patients carrying this missense variant is very similar to that of the carriers of the other truncating variants. Interestingly, functional studies on the mutant models suggested that while the binding affinity of LIMP-2 to GC has been perturbed in loss of function variant carriers (p.W146SfsX16, p.W178X), it has been conserved in the missense variant carrier (p.H363N) as well as in one particular loss of function variant carrier (p.Q288X), where the essential binding site is preserved [6]. However, even though the binding property of LIMP-2 to GC has been shown to differ based on the type of the variant, the ER retention of LIMP-2 has been reported to be common in all four investigated variants [6]*.* These findings suggest that other mechanisms or modifiers might play a role in the pathogenicity of the disease and lead to a complex pathogenic mechanism. One of those possible implicated mechanisms is that progranulin couples heatshock-protein 70 (HSP70) to the GCase/LIMP2 complex in the ER and leads the cargo of the GC to the lysosomes [36] (Fig. 2–2).

The majority of the reported pathogenic variants are localized in the luminal domain except for the two variants (p.L14PfsX35, p.G462DfsX34) which are localized in the TM domains. One of those (p.G462DfsX34) is reported to cause a milder phenotype with a later AO and the absence of renal involvement in three patients from two unrelated families originating from Japan[17, 18] (Table 2, Fig. 2–1). In addition, this variant has been found to be significantly associated with a higher AO. Compared to the carriers of other variants the difference of the p.G462DfsX34 phenotype has been attributed to the residual activity of the protein [17]. The other pathogenic variant (p.L14PfsX35) is localized in the N terminal site of the protein, and it is expected to cause a total loss of the luminal and C terminal TM domains. Therefore, its effect on the clinical severity is similar to the clinical presentation of patients carrying variants in the lumenal domain.

Clinical characteristics of the reported pathogenic variant carriers have been shown to be common features such as myoclonus, ataxia, tremor, dysarthria, tonic clonic seizures, renal impairment and more rarely observed uncommon features including PNP, hearing loss, and cognitive impairment (Table 2). Interestingly, among those clinical features, cognitive impairment has only been shown in three particular variant carriers (p.G462DfsX34, p.R121X, p.N45MfsX88) originating from Japan and Turkey [17, 18]. Therefore, it is important to note that even the patients do not complain about cognitive functions, a detailed cognitive testing is required to detect subtle cognitive deficit in the individuals with AMRF. Of those clinical characteristics PNP and hearing loss have been reported in the knockout animal models [37].

Strikingly, some SCARB2 variants have not been directly associated with AMRF (Table 3). Of those variants the heterozygous missense variants have been reported to be implicated in PS, GD, ASD and sudden unexpected death in epilepsy, suggesting the role of SCARB2 gene in those disease mechanisms. Even though some of the reported biallelic pathogenic variants have not been reported to be associated with AMRF, given the observed clinical characteristics carriers of those might have been clinically misdiagnosed.

Symptomatic therapeutic interventions for the AMRF include targeting the neurological and renal manifestations. In general, Valproic acid, Clonazepam and Levetiracetam are administered against the neurological manifestations, dialysis and renal transplantation performed against the renal manifestations. Miglustat as a new therapeutic approach, which is an inhibitor of glucosylceramide synthase, has also been experienced in the clinical practice. However, even though miglustat treatment has been reported to be effective by reducing the myoclonic jerks in a patient with AMRF by Chaves et al. [38] and by improving dysphagia and other associated symptoms without any side effects by Quraishi et al. [27], other independent studies suggested that the treatment caused several side effects, such as nausea, vomiting and severe diarrhea [9, 25] and was therefore discontinued. Miglustat treatment was also administered to our two affected individuals (family1; II.8, II.12). Both patients could also not tolerate miglustat even at lower doses, due to severe exacerbation of action myoclonus and ataxia. The siblings with mild neurological presentation (family 1; II.10, II.13) unfortunately refused to start miglustat treatment in early stage of the disease, and no information about a possible positive effect on disease progression could be evaluated. However, given the reported number of patients not tolerating this treatment, further administration needs to be planned carefully.

Mainly renal manifestations of SCARB2 associated clinical picture include FSGS, or membranous nephropathy without storage [1, 25, 39]. Treatment of renal failure in AMRF patients is supportive. Renin-angiotensin blockage can prevent hyperfiltration, and proteinuria, and dialysis treatment is supposed to extend the life expectancy [39, 40], however, the neurological findings do not improve with dialysis. In our study the renin-angiotensin blockage decreased the proteinuria and slowed the progression of the end-stage renal disease in the individual II.8.

In our study WES analysis revealed a homozygous truncating variant (c.134delA**;** p.N45MfsX88) in the index case and subsequent sanger sequencing analysis confirmed the co-segregation of the variant with the disease. This is the second report describing the same pathogenic variant (c.134delA**;** p.N45MfsX88) in SCARB2 gene from Turkey, thus suggesting a possible founder affect. As a result of comparison with the family 2, intra and inter-familial clinical heterogeneity was observed with some common features with slightly different AO and distinct features, such as dysarthria and cognitive decline.

Similar clinical heterogeneity with a wide spectrum of neurological and renal symptoms was observed in all SCARB2 pathogenic variant carriers. Among the reported variants only the one (p.G462DfsX34) localized in the C terminal site of the LIMP-2 has been statistically found to be associated with later onset of the disease.

*SCARB2* is an important gene with an essential function in pathogenic mechanisms that are important in AMRF, GD, and PD. Therefore, genetic and functional studies to understand common and distinct mechanisms underlying all those three diseases have a great importance. So far it has been suggested that disrupted binding of LIMP-2 and GC does not necessarily have an impact on the disease pathogenesis and GC activity might be different in different cells probably based on the expression levels of the genes or the effect of the different modifiers. (Fig. 2). Nevertheless, further questions such as, which modifiers play a role in those pathways (Fig. 2) and why only some of the specific systems, such as brain and kidney, are particularly vulnerable (Fig. 2), remain elusive. Therefore, patients-specific induced pluripotent stem cell-based studies might be in the future the most promising way to contribute the best to the understanding of the *SCARB2* related pathology in the related disease mechanisms.

## Supplementary Information

**Additional file 1.**

**Additional file 2. **

## Data Availability

The datasets generated during and/or analysed during the current study are available from the corresponding author on reasonable request. The detailed information regarding the identified variant is available in the ClinVar repository under the accession number of SCV002097583.

## Abbreviations

AMRF: Action myoclonus-renal failure syndrome
WES: Whole exome sequencing
AO: Age at onset
PD: Parkinson’s disease
GD: Gaucher disease
SCARB2: Scavenger receptor class B member 2
LIMP-2: Lysosomal integral membrane protein type 2
GC: β-Glucocerebrosidase
ER: Endoplasmic reticulum
MRI: Magnetic resonance imaging
NCV: Nerve conduction velocity
SEP: Somatosensory evoked potentials
EEG: Electroencephalography
FSGS: Focal segmental glomerulosclerosis
EMG: Concentric needle electromyography
PNP: Peripheral neuropathy
CT: Cytoplasmic
TM: Transmembrane
